# ASSESSING THE PREDICTIVE POWER OF VOLUNTEER FREQUENCY CATEGORIES ON HEALTH: AN OUTCOME-WIDE APPROACH

**DOI:** 10.1093/geroni/igae098.4120

**Published:** 2024-12-31

**Authors:** Samuel Nemeth, Seoyoun Kim, Cal Halvorsen, Isha Patel

**Affiliations:** Rutgers School of Public Health, Piscataway, New Jersey, United States; Texas State University, San Marcos, Texas, United States; Washington University in St. Louis, St. Louis, Missouri, United States; Texas State University, San Marcos, Texas, United States

## Abstract

Later life volunteering contributes not only to community betterment, but also significant health benefits for volunteers themselves. However, researchers have used various methods for measuring volunteering frequency—often with data from the Health and Retirement Study (HRS)—ranging from binary to eight-level categories. These diverse categorizations can obscure an accurate understanding of the relationship between volunteering and health while causing confusion among authors and reviewers on the ideal way to specify volunteering frequency. In response, we conducted a longitudinal outcome-wide analysis using nationally representative panel data from the HRS from 2006-2016 (N=17,062). We examined five different categorizations (2, 3, 4, 5, and 8 categories) of volunteering frequency at “baseline” (i.e., 2012, 2014) to examine how they differentially predicted 12 health outcomes (e.g., self-rated health, depressive symptoms, blood pressure, cholesterol) in the following wave (i.e., 2014, 2016). We found that as the number of levels of volunteering increased, the estimated effects on health become more attenuated (i.e., the effect sizes reduced). However, the confidence intervals become increasingly tighter, indicating that multiple categories of volunteering explain a larger proportion of variance in health outcomes more effectively. Importantly, and regardless of the measurement strategy used, the substantive conclusions for the health benefits of volunteering did not change. Rather than recommending a “best” practice, we advocate for a carefully reasoned selection of volunteering categorizations when designing studies and interpreting results that most optimally apply to researchers’ objectives.

